# TeachMe: a web-based teaching system for annotating abdominal lymph nodes

**DOI:** 10.1038/s41598-022-08958-8

**Published:** 2022-03-25

**Authors:** Shuaihua Chen, Hao Huang, Xuyang Yang, Han Wang, Mingtian Wei, Haixian Zhang, Ziqiang Wang, Zhang Yi

**Affiliations:** 1 Machine Intelligence Laboratory, College of Computer Science, Sichuan University, Chengdu, 610065 People’s Republic of China; 2 Gastrointestinal Surgery Center, West China Hospital, Sichuan University, Chengdu, 610041 People’s Republic of China

**Keywords:** Medical imaging, Gastroenterology

## Abstract

The detection and characterization of lymph nodes through interpreting abdominal medical images are significant for diagnosing and treating colorectal cancer recurrence. However, interpreting abdominal medical images manually is labor-intensive and time-consuming. The related radiology education has many limitations as well. In this context, we seek to build an extensive collection of abdominal medical images with ground truth labels for lymph nodes recognition research and help junior doctors to train their interpretation skills. Therefore, we develop TeachMe, which is a web-based teaching system for annotating abdominal lymph nodes. The system has a three-level annotation-review workflow to construct an expert database of abdominal lymph nodes and a feedback mechanism helping junior doctors to learn the tricks of interpreting abdominal medical images. TeachMe’s functionalities make itself stand out against other platforms. To validate these functionalities, we invite a medical team from Gastrointestinal Surgery Center, West China Hospital, to participate in the data collection workflow and experience the feedback mechanism. With the help of TeachMe, an expert dataset of abdominal lymph nodes has been created and an automated detection model for abdominal lymph nodes with incredible performances has been proposed. Moreover, through three rounds of practicing via TeachMe, our junior doctors’ interpretation skills have been improved.

## Introduction

Colorectal cancer (CRC) is one of the most common causes of cancer death in China and many other countries around the world^1–3^. To prevent CRC recurrence and achieve the best prognostic, the detection of abdominal lymph nodes (LNs) is of great importance^4–6^. Generally, contrast-enhanced computed tomography (CE-CT) scan of the whole abdomen is a crucial means to identify whether abdominal LNs have metastasis and diagnose CRC. However, there are many limitations when interpreting CE-CTs manually. Firstly, misdiagnosis and missed diagnosis often happen, which dramatically delays the golden time limit for treatment. Besides, it will take a considerable labor cost if all interpretations need to be done by professional senior doctors. Moreover, qualified doctors are limited in some rural areas, for which reason many patients have difficulties seeing a doctor in the early stage of the disease. To address the problem presented above, medical schools must strengthen personnel training to improve junior doctors’ professional quality. As for medical education, radiology education is a vital part of the undergraduate medical curricula. It is all about the essential skills to diagnose CRC and other diseases. Worse still, radiology education for junior doctors is also limited at present. Since the skills of interpreting medical images rely significantly on experience, junior doctors need much training to accumulate knowledge to achieve an expert level. But they are often time-poor as they need to balance clinical practice with medical theory learning and other responsibilities. Many of them can not get enough practice at school consequently. Still, their learning outcomes sometimes are not assessed by their tutors in time. Therefore, to promote radiology education in medical school, it is necessary to improve the traditional teaching way in class and create a new teaching method. Furthermore, to solve the limitations in diagnosing CRC fundamentally, creating a support to assist doctors in interpreting medical images is in demand as well. With the rapid development of artificial intelligence and Internet technology, a computer-aided system may provide an outlet for these issues. For one thing, previous studies^7–9^ have proved that deep neural networks have had tremendous success in solving medical problems over the past few years. It suggests that trianing an accuracy neural network model using a large dataset with precise labels is an effective way to reduce doctors’ working intensity and improve the efficiency. For another, previous studies^10^ have demonstrated the efficacy of radiology-themed online e-learning platforms for senior medical students. Those e-learning platforms provide a more flexible learning style for junior doctors. Motivated by the potential, we intend to develop a computer-aided system to collect an expert dataset of abdominal lymph nodes supporting the research of automated lymph nodes detection models and assist junior doctors to practice their interpretation skills. With this purpose, we do the following analysis and investigation.

**Figure 1:**
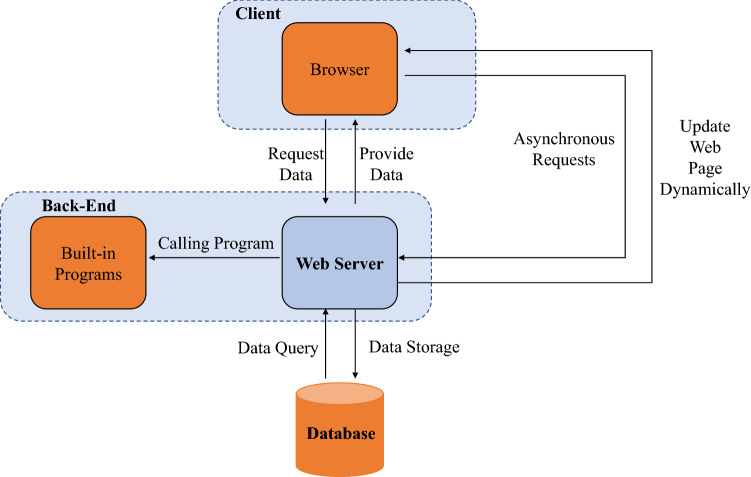
The browser/server architecture of TeachMe.

**Figure 2:**
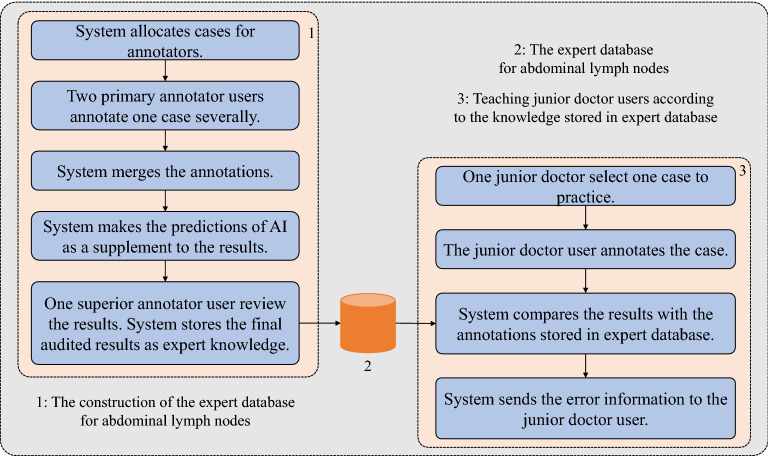
The three functional modules and their workflow of TeachMe. Module 1 is the construction of the expert database for abdominal lymph nodes, which involves the primary annotator users and the superior annotator users. Module 2 is the expert database based on MongoDB. Module 3 is the feedback mechanism, which is the teaching process in TeachMe by comparing the “answers” of junior doctor users with the knowledge in the expert database.

**Figure 3:**
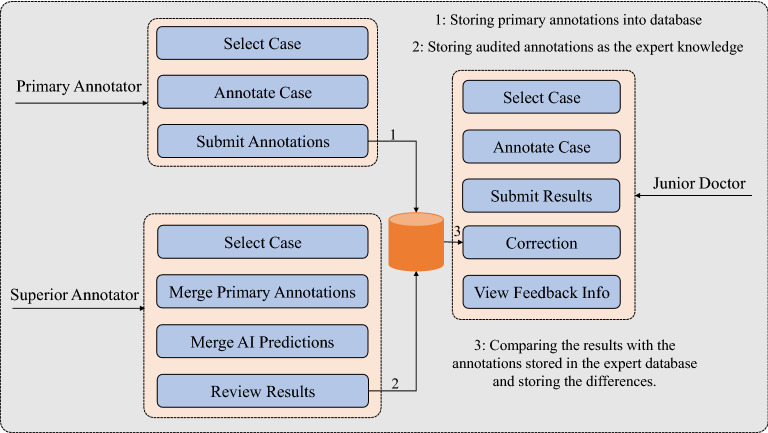
The overview of the three user interfaces. TeachMe provides different functionality for different types of users. The primary annotator users and the superior annotator users take part in the construction of the expert database. The primary annotator users are responsible for giving the primary annotations. The superior annotator users make the final review and confirmation. The annotations audited by superior annotator users are the expert knowledge stored in the expert database. The junior doctor users are the service object of the constructed expert database. TeachMe can correct their submitted “answers” owing to the feedback mechanism of TeachMe.

To start with, what matters first for constructing a dataset of abdominal lymph nodes is a convenient annotating tool for doctors to cooperate with the collection process. Nevertheless, the existing annotating tools are not proper to satisfy the need. Generally, the annotating tools used in hospitals are the customized system. Most of them are desktop applications, which must be part of the hospital’s network to gain direct access to the picture archiving and communication systems (PACS). The networking requirement reduces efficiency and limits flexibility. Though there are some other informal annotation tools, such as LabelMe^11,12^, ITK-SNAP^13^ and 3D-Slicer^14^, they are still not appropriate to annotate abdominal lymph nodes. LabelMe^11,12^ is a web-based annotating tool providing polygon labeling and other functions. But it aims to annotate natural images and is unsuitable for medical images. ITK-SNAP^13^ and 3D-Slicer^14^ can browse and annotate DICOM (Digital Imaging and Communications in Medicine) images. They also provide much functionality, such as medical image visualization, manual segmentation, and semi-automatic segmentation. However, they do not provide a unified standard of annotations and cannot centrally store the annotation information, which are not convenient for several annotators to collaborate with each other. On the other hand, we have investigated the existing e-learning platforms of radiology education and not found an interactive platform specializing in training interpretation skills for abdominal CE-CTs either. As far as a great teaching platform’s concerned, one of the most critical elements is the platform’s content provided to the students. Since our purpose is to construct a platform for junior doctors to practice specialized skills in detecting abdominal lymph nodes through CE-CTs, the content must be very reliable and advanced. Furthermore, to achieve better teaching effect, the platform should be interactive. However, the following six existing e-learning platforms are not perfect. Firstly, MyPACS.net^15^ and COMPARE Radiology^16^ are web-based authoring tools that teachers can create teaching content using medical images. But both of them only focus on passive theoretical information delivery, not providing functionalities for students to practice skills. Though ELERA^17^ and Radiology ExamWeb^18^ can provide the functionalities to create tests or give the learners guided instruction and feedback, they still focus on the basic concepts, not the expert diagnosis skills. Similarly, RadStax^19^ and RadEd^20^ can create labels on regions of interest of the medical image and teach how to detect them. But the former is only concerned with introducing information related to these labels, and the latter’s teaching content is relatively simple. All in all, these existing e-learning platforms are online classrooms essentially but not clinical practice tutorials. They are not the appropriate platforms for training the skills of abdominal lymph nodes detection, which are complicated and tricky.

Based on the points discussed above, this paper introduces TeachMe. TeachMe is not just an annotation tool for annotating abdominal lymph nodes but also a teaching platform providing instruction on practicing abdominal medical images interpretation skills. Junior doctors can conduct specified training of interpretation skills in this system. They can advance their interpretation skills by repeatedly practicing via this system with error informations provided. Thus, the goals of TeachMe are summarized as follows:

1. To provide annotating functionality for making accurate annotations on abdominal CE-CTs;
2. To archive the precise locations and labels of the annotations;
3. To interact with junior doctors and help them improve their interpretation skills for detecting abdominal lymph nodes.

To achieve these goals, TeachMe is developed as a web-based teaching system for annotating abdominal lymph nodes with three main modules: a construction process of the expert database for abdominal lymph nodes, an expert database for abdominal lymph nodes, and a feedback mechanism providing the corrections for junior doctor users. To construct the expert databse, TeachMe adopts a three-level annotation-review workflow to collect the annotated data with the gold standard and ensure the expert database’s accuracy. The annotations of abdominal lymph nodes stored in the database and the stored information can be exported as a traning dataset easily. TeachMe can teach junior doctors via the feedback mechanism using the stored expert content, which is the most significant difference between TeachMe and other platforms and makes itself stand out as a novel teaching system. To validate TeachMe’s functionalities, several gastroenterology doctors in our medical team have used the system, from whom a dataset of abdominal CE-CTs with accurate lymph nodes annotations called PLNDataset has been created. With the dataset, an effective neural network model for detecting abdominal lymph nodes has been proposed in^21^. Moreover, with TeachMe’s feedback mechanism, the junior doctors in our medical team has improved their interpretation skills through three rounds of practicing. In this work, the proposed TeachMe provides functionalities to collect an expert dataset of abdominal lymph nodes with the gold standard and tutor junior doctors according to the constructed expert knowledge. The development of TeachMe is the critical first step to promote the research of interpreting CE-CTs automatically and solve the limitations from clinical work. More importantly, it provides a novel way to advance radiology education for junior doctors.

## System description

This section presents TeachMe’s architecture with its main modules and its functionalities. Firstly, the architecture of TeachMe is shown in Fig. 1. The main components of the architecture are the client, the back-end, and the database. The client provides functionalities that are realized in the user interfaces through web browsers. The back-end processes users’ requests from the client. And the database stores all annotations with gold standards and other related information. To get the web page implemented with HTML (the HyperText Markup Language), the client sends requests to the back-end. The web server based on Node.js handles and responds to these requests, providing related data to the client. Moreover, some asynchronous requests realized with AJAX (Asynchronous JavaScript and XML) are sent to the server to retrieve data in JSON (JavaScript Object Notation) format and update the HTML web pages dynamically, without reloading the entire page. As for the requests handling process, the server queries the requested data from the database and provides the processed data to the client. Besides, some functionalities provided by TeachMe are implemented with Python. So the server will call the related built-in programs to realize these functionalities once the client sends the corresponding request to the back-end. Overall, the key technologies to realize TeachMe’s architecture are summarized as follows: TeachMe follows a Browser/Server architecture (described in Fig. 1), which is an effective improvement of the Client/Server architecture with the development of Internet technology. The web server is based on Node.js, which is an open-source, cross-platform, back-end JavaScript runtime environment. And the database is based on MongoDB, which is a non-relational database with great performance and expansibility. To implement the interfaces and the application’s logic, we used HTML5 and JavaScript, respectively. Moreover, we use Python to write corresponding scripts realizing some functionalities of the back-end, such as merging annotations and the feedback mechanism. As for the development environment, we deploy TeachMe using tmux on the server with a Linux operating system. Since TeachMe follows the Browser/Server architecture, TeachMe and its functionality will be delivered to client machines exclusively through a web browser such as Chrome and Mozilla Firefox.

Secondly, Fig. 2 describes the platform’s main modules and their functionalities. There are three main modules integrated into TeachMe. The first is the annotating process to construct an expert database, the second is the expert database itself, and the third is a feedback mechanism for correction. All of them are indispensable for teaching purposes. Especially, the feedback mechanism is established on the basis of the constructed expert database because the system computes the feedback information according to the content in the expert database. Thus, constructing the expert database is the first step. Once one case gets annotated and its annotations get reviewed, the expert knowledge of the case will be stored and used for correcting junior doctors’ annotations as the “answers”. Thirdly, as shown in Fig. 3, the platform supports three user types: primary annotator users, superior annotator users, and junior doctor users. TeachMe provides different functionality for them. Both primary annotator users and superior annotator users participate in the construction of the expert database for abdominal lymph nodes. The primary annotators are responsible for submitting the preliminary annotation results. The superior annotators are responsible for reviewing and confirming the final results. In contrast, junior doctor users are the service object of the system. The system serves the junior doctor users based on the knowledge in the expert database. They are just like the examinees who take part in an exam of detecting abdominal lymph nodes. So they are welcome to give their answers as many as they can. The system will make corrections by comparing the results with the annotations stored in the expert database. To introduce more details, the following subsections present TeachMe’s three main modules, respectively.

### Three-level annotation-review workflow

The three-level annotation-review workflow is realized by the human experts and a well-trained automoted detection model together. They work together to construct the expert database of abdominal lymph nodes. Human experts refer to primary annotators and superior annotators. First and foremost, paired primary annotators annotate a given CT study independently. When they finish the annotations, TeachMe merges the same annotations and keeps the different annotations of the paired primary annotators. Next, the system marks the annotators’ names for each annotation as a signature. Then TeachMe stores the annotation information, creating the prototype of the expert database. Based on this prototype, TeachMe compares the predictions of the automated detection model with the first-level annotation results to obtain the candidates of lymph nodes that all first-level annotators have missed. In the end, a given superior annotator will check the annotations that have been calibrated by the model and make corrections. They need to confirm if there are incorrect annotations or omitted lymph nodes. Thus, the accuracy of the expert database is guaranteed by the workflow. Through the three-level annotation-review workflow, TeachMe can construct an expert database containing precise annotations of abdominal lymph nodes.

### Expert database of abdominal lymph node

Apart from the information of locations and classifications of lymph nodes, the expert database includes other essential details. For one thing, the annotated start slice and end slice are important parts of the expert knowledge. The start slice refers to the frame when the aorta starts to separate to the right common iliac artery and left common iliac artery. And the end slice refers to the section when the symphysis pubis part starts to bifurcate. For another thing, TeachMe labels a specific index based on a particular sorting rule for all annotations. Every time annotators create a new annotation, TeachMe will calculate its index according to its location and classification. Here is the sorting rule: if there is more than one annotated region of the same type almost at the same horizontal level on one slice, the order of these annotations will ascend from left to right breadthways; If there is more than one annotated region of the same type, almost at the same vertical level on one slice, the order of these annotations will be descending bottom to top lengthways. Besides, the signatures of annotations are also important information of these annotations.

### Correction for junior doctors’ annotation results

When a junior doctor user uses TeachMe to practice the skills of detecting abdominal lymph nodes, he/she should choose a study, view the study frame by frame and make annotations as much as possible by oneself. When he/she submits the final results, TeachMe will compare the results with the gold standard knowledge stored in the expert database. Every time a junior doctor user finish an exercise, TeachMe will analyze and record the neglected region and the wrong labeled region. To analyze the annotation results, TeachMe excludes the missed annotations and the wrong labeled annotations, so the rest annotation regions are the correct regions included in the gold standard annotations. Then TeachMe assesses if the rest annotations’ classification labels are accurate. After this process, TeachMe replies to the junior doctor with a report about how to correct his/her annotations. Thus, junior doctors can improve their ability to detect the abdominal lymph nodes through the annotation practice in TeachMe again and again. What is more, TeachMe can also reply with the correct start slice and the end slice if the junior doctor has labeled them wrong.

## User interfaces of TeachMe

**Figure 4:**
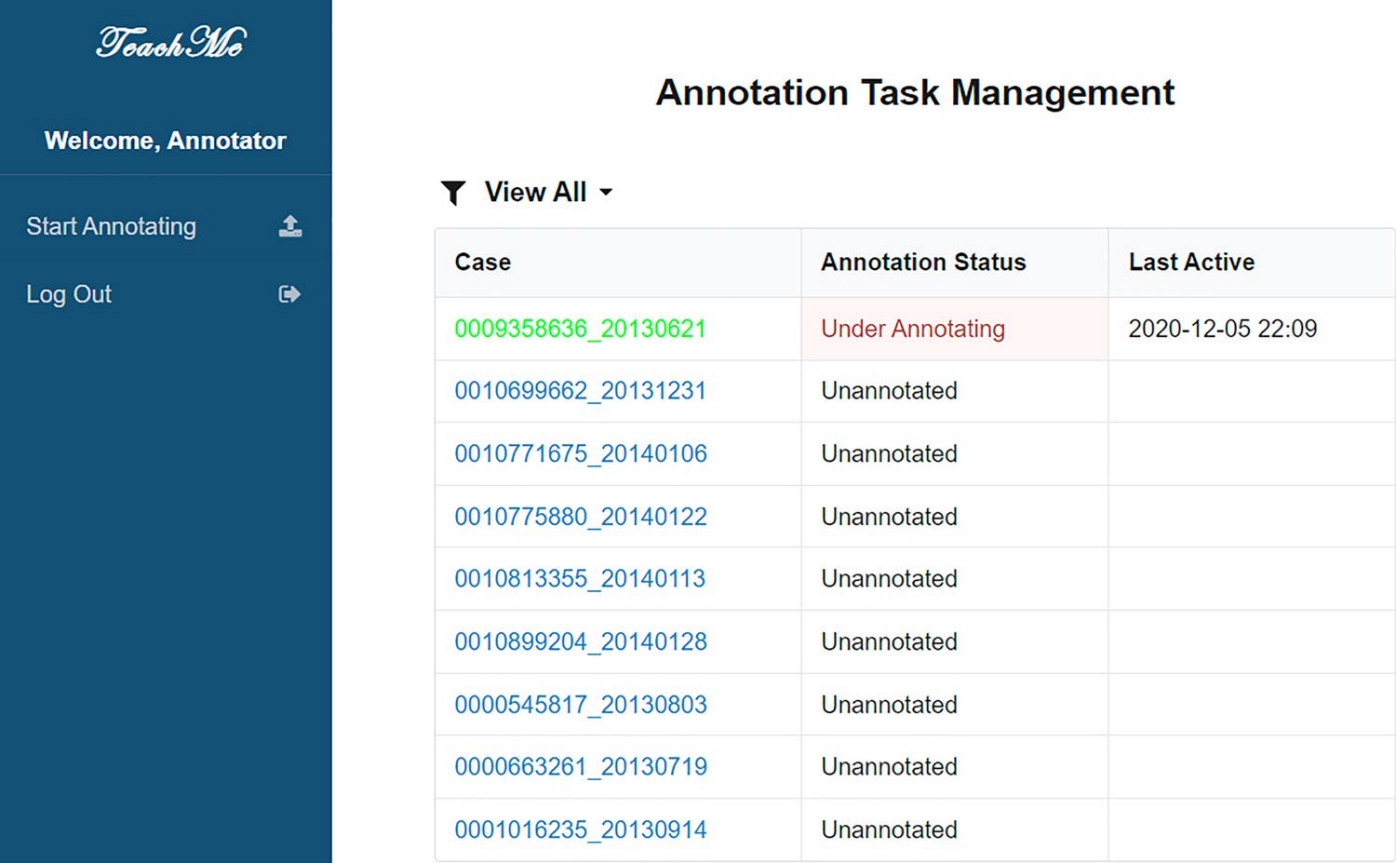
The annotating tasks management interface.

**Figure 5:**
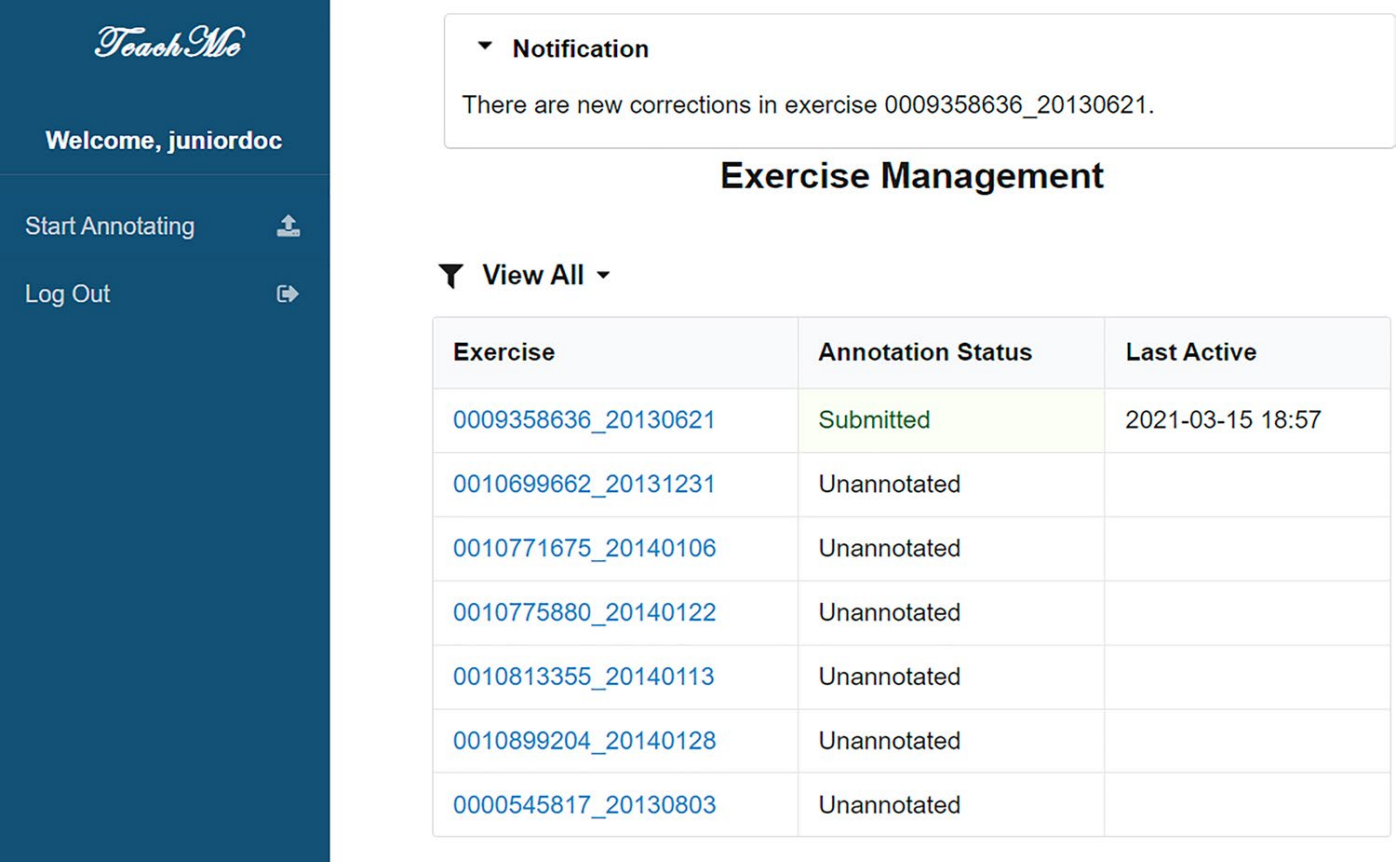
The exercises management interface of junior doctor users.

There are two parts of the user interface (UI) in TeachMe: the UI of management of annotation tasks and the UI of annotation canvas. Moreover, there are three main roles in TeachMe: primary annotators users, superior annotators users, and junior doctor users. Figure 4 shows the annotation task management page. The reviewing task management page approximately the same, listing the table of annotation/review studies the annotator needs to annotate. Annotators can click the corresponding study and enter the annotating/reviewing page. The junior doctor users’ annotation task management page slightly differs as shown in Fig. 5. It has the dialog box indicating which exercise TeachMe has corrected. The junior doctor user can click the corresponding one to enter into the viewing page and check. The UI of the annotation canvas roughly the same.

**Figure 6:**
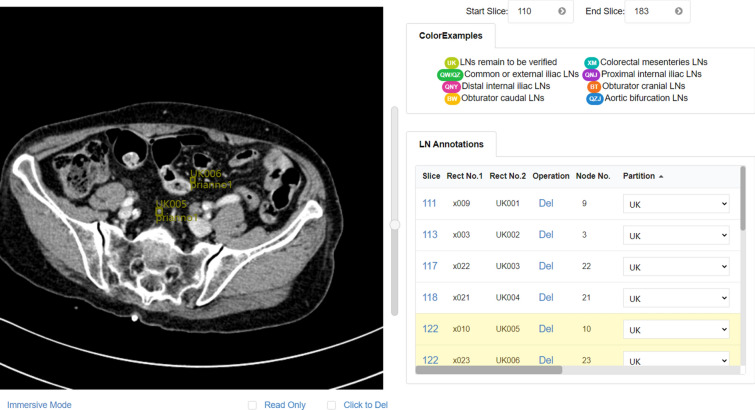
The annotating/reviewing page of the working interface (zoom mode and editable).

Figure 6 illustrates the annotating/reviewing page. The annotation canvas is on the left, displaying the medical images of the selected study. Users create all annotations on the canvas. Users can scroll the upright progress bar to slide the CT images and get a quick look through. When users find out an abdominal lymph node, they just need to drag the mouse drawing a box and the region will be annotated. If the annotation is not proper, they can remove the box to modify the locations or the range or delete it directly. TeachMe stores the coordinates of the annotation in real-time. The annotations table on the right demonstrates the detailed information of annotated lymph nodes. The detailed information includes the index of the annotation, the sequence number of the CT slice on the axis plane, and the classification labels. TeachMe provides autosave functionality. The expert database based on MongoDB archives all information of annotated abdominal lymph nodes.

**Figure 7:**
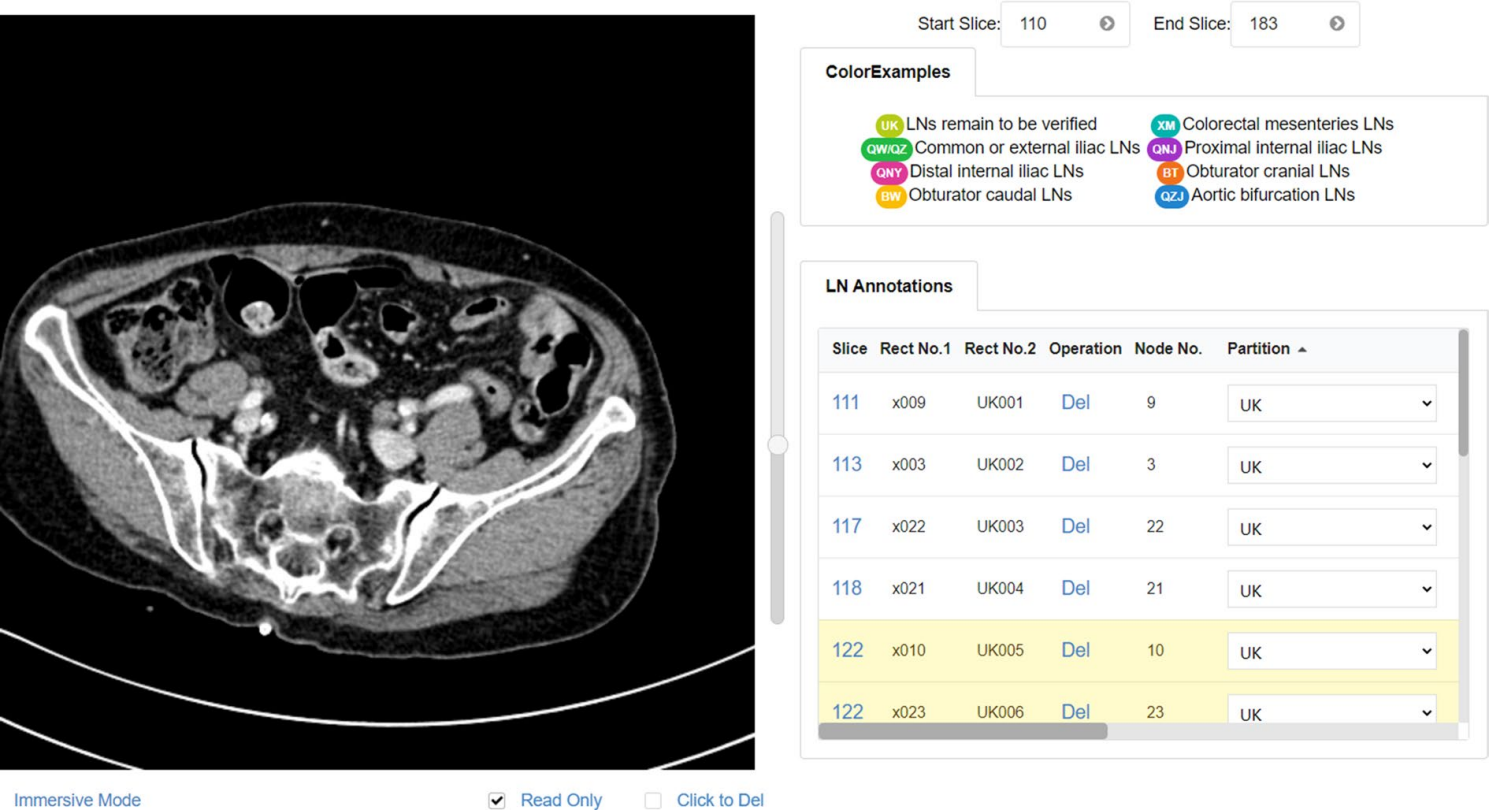
Zoom mode and read only.

**Figure 8:**
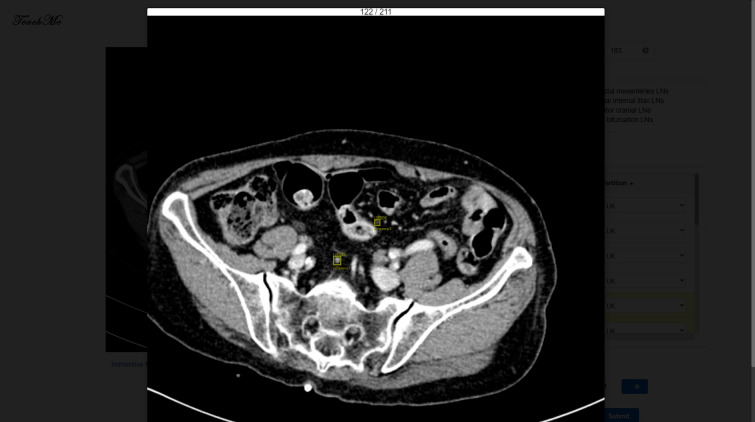
Immersive mode and editable.

**Figure 9:**
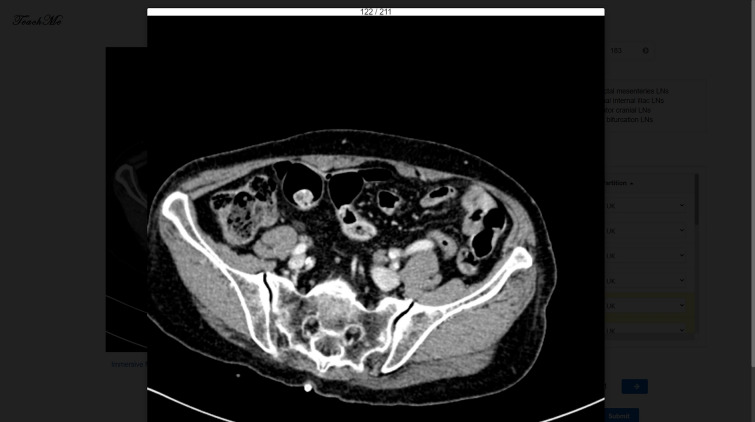
Immersive mode and read only.

There are the Zoom Mode and the Immersive Mode of the canvas. TeachMe magnifies the original image 1.5 times in the Zoom Mode. It is convenient for users to interact with the annotation table in this mode. In the Immersive Mode, TeachMe magnifies the original image three times. Users can detect the abdominal lymph nodes easier due to a larger vision. Besides, there are the Read-Only Mode and the Editable Mode. The Read-Only Mode intends not to disturb the view of annotators, especially when there are many annotated ROIs (Region of Interest). In the Read-Only Mode, the canvas does not show the annotated ROIs. Users can not draw ROIs in this mode, providing clean medical images for annotators viewing. The Editable Mode is on the contrary. Figures 6, 7,  8 and  9 show these four modes respectively.

**Figure 10:**
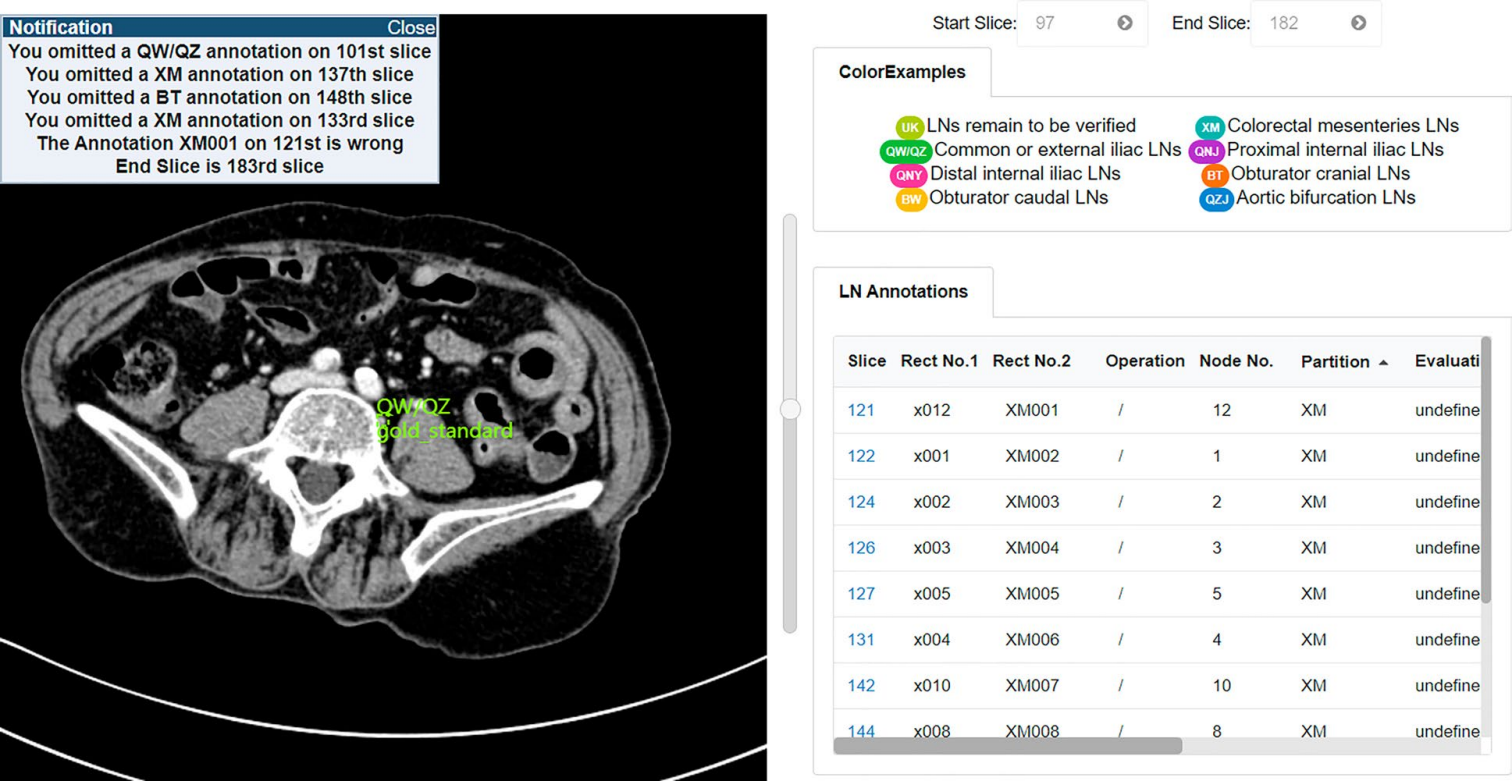
The annotating page of the working interface of junior doctor users.

Figure 10 shows the annotating page of the working interface. The dialog box on Fig. 10 shows the detailed correction information for the junior doctor user. Each line in the dialog box displays a message of one annotation, and when the junior doctor user clicks one of them, TeachMe will highlight the corresponding incorrect annotation. The correction information includes which annotations the junior doctor omitted, mistaken, and not quite right. For the record, the omitted annotations refer to those lymph nodes not annotated by the junior doctor user but exist in the gold standard. The incorrect annotations refer to the annotated region that the junior doctor user drew that does not contain gold standard lymph nodes. And the annotations not quite right refer to the annotations’ labels are wrong compared with the gold standard.

## Results and discussions

### User impressions

**Figure 11:**
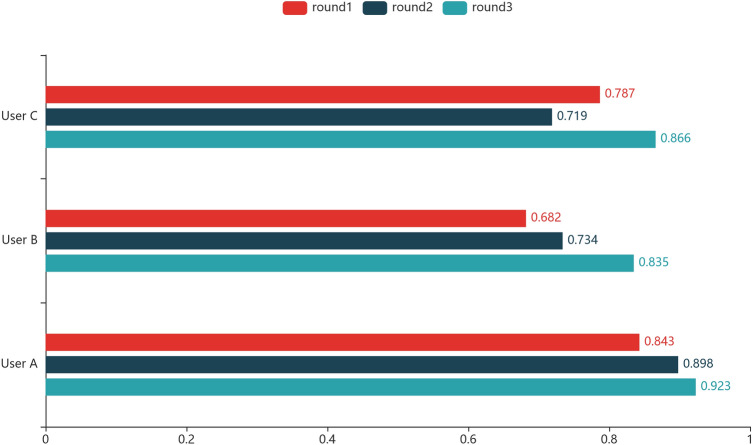
Accuracy of junior doctor users in three rounds exercise.

To collect the dataset of abdominal lymph nodes and construct the expert database, we finish three rounds of data collection with the participation of gastroenterology doctors from West China Hospital. We invite six PhDs as primary annotators and two gastroenterology experts as superior annotators. They participated in the annotation-review workflow and finished the first round of data collection, creating a dataset consisting 103 CE-CT studies with 2171 abdominal lymph nodes annotated and reviewed. Then, we use the collected dataset to construct a small-scale expert database and train a neural network model. In the subsequent two rounds of data collection, the neural network model started participating in the annotation-review workflow supporting the doctors. In the end, a large-scale of dataset has been created successfully, which is introduced in subsequent subsection. Besides, to test the feedback mechanism of TeachMe, we invite three undergraduates in our medical team to annotate different assignments independently. When they finish each of the studies, TeachMe will contrast their annotations with the “answer” stored in the expert database and provide them immediate feedback, showing them the error information. Long-term exercising and error correction allow them to discover their areas of weakness and come to understand what they should concentrate on when restarting new exercising. As shown in Fig. 11, two users’ accuracy of detecting the abdominal lymph nodes has improved significantly after three rounds of exercises. Though user C got his worst performance in the second round, the difference in his accuracy between the first two rounds is not significant and in a normal range. The possible reason for this situation is that the junior doctor’s state fluctuated at that time affected by some disturbances. Moreover, after using TeachMe for a period of time, all doctors expressed their impressions on the platform. All of them considered TeachMe is practical and easy to use. For the annotators, they think TeachMe is valuable to collect medical datasets and helpful for their medical research. For the junior doctors, they agree that TeachMe can tell them some tiny lymph nodes that they should have detected but omitted and it does help them to improve their skills.

### Comparison with other systems

The following features distinguish TeachMe from other annotating tools:

1. Three-level annotation-review workflow to ensure the accuracy of the expert database; Primary annotators annotate each study first. A neural network model successively supplies candidates omitted. Superior annotators confirm the annotations at last. The three-level annotation-review workflow plays a role as a peer-review mechanism to guarantee the content’s reliability.
2. A Built-in unified sorting rule for annotation; Each annotation has a given serial number based on a specific law. It is convenient for doctors to track the lymph nodes for the follow-up study. Moreover, it provides a straightforward way of referring to a specific lymph node during a consultation.
3. A feedback mechanism to make assessment and interact with junior doctors; TeachMe can make assessments and reply to the junior doctor users with the corrections of annotations. It is convenient for junior doctors to discover their weakness and improve their interpretation skills.

In Table 1, we compare TeachMe with other platforms we mentioned in the Introduction. Equipped with the three-level annotation-review workflow to peer-review the teaching content, a unified annotating rule to guarantee teaching content’s consistency and a feedback mechanism to make assessments, TeachMe is more professional as a teaching platform for practice advanced clinical skills. Thus, more considerate functionalities make TeachMe stand out.

**Table 1:** Comparison of main web-based systems for radiology education.

	MyPACS ^15^	COMPARE ^16^	ELERA ^17^	ExamWeb ^18^	RadStax ^19^	RadEd ^20^	TeachMe
Teaching content	Theory	Theory	Theory	Theory	Annotation (basic)	Annotation (basic)	Annotation (advanced)
Peer-review workflow for teaching content	No	No	No	No	No	No	Yes
A unified rule for annotation	No	No	No	No	No	No	Yes
Assessment strategies	No	No	No	Yes	No	Yes	Yes

### Dataset and model

**Figure 12:**
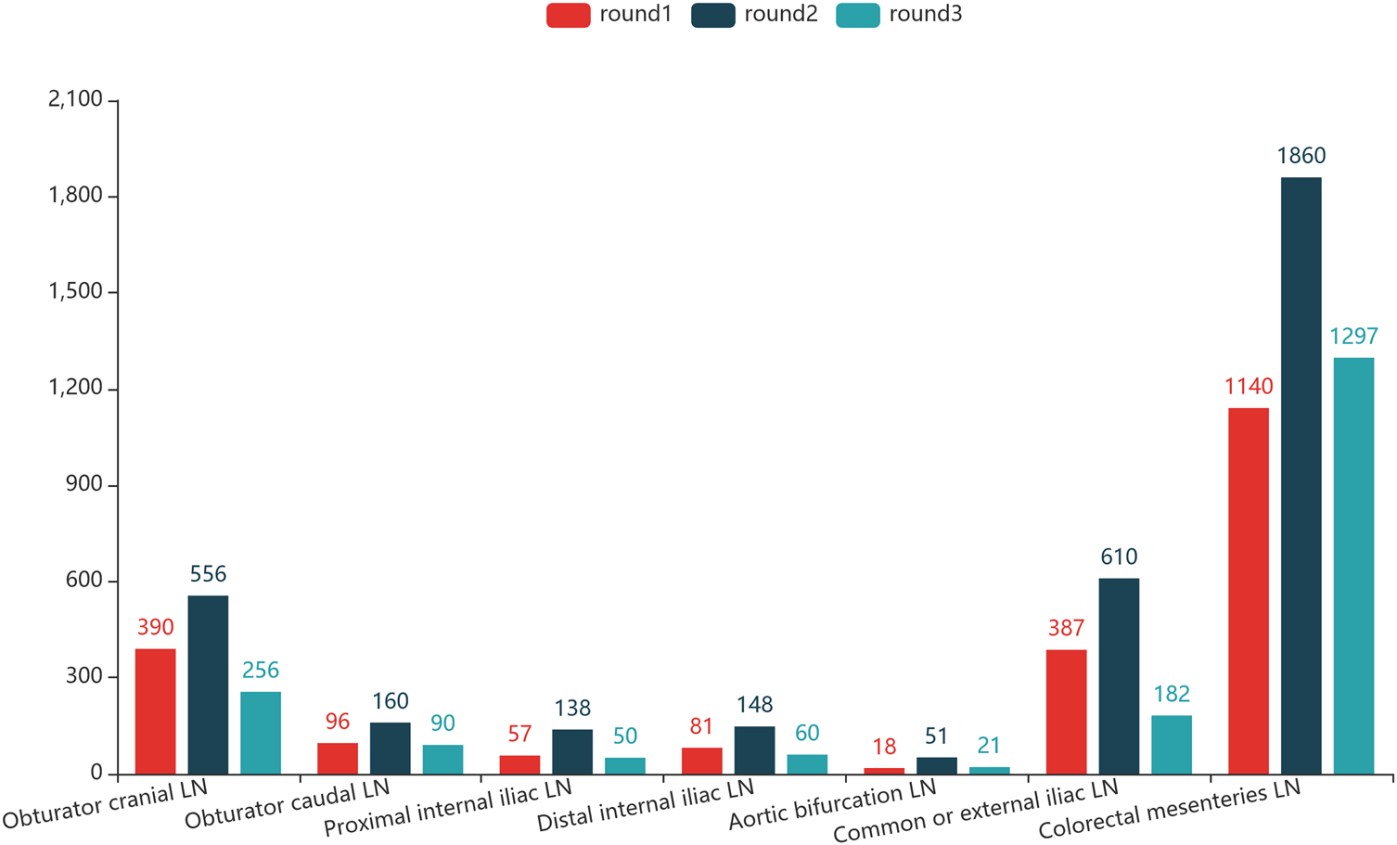
Partition distribution of annotated lymph nodes.

After three rounds of data collection, PLNDataset is constructed. It contains 236 CRC patients’ CE-CT with 6880 abdominal lymph nodes’ position and classification information in total. As mentioned above, the 6880 abdominal pelvic lymph nodes’ annotations have been made and checked strictly by professional gastroenterology doctors via the three-level annotation-review workflow. After annotators draw the bounding box of the lymph node using the annotation tool in TeachMe, they will select the label to which the lymph nodes’ region belongs. After careful consideration, our gastroenterology experts decided to divide the abdominal pelvic lymph nodes into seven regions, which we call the seven partitions of abdominal lymph nodes. Figure 12 illustrates the partition distribution of annotated lymph nodes in the three rounds of data collection. These information plus the pelvic region’s start and end frame are the final expert knowledge of abdominal lymph nodes collected by TeachMe. With these knowledge, more valuable research can be conducted. We propose an automated lymph node detection method in^21^ PLNDataset. The proposed method uses the spatial prior knowledge to detect the pelvic region on the CE-CT images at the first stage. Then, a neural network model with two main pathways is applied to detect and locate lymph nodes all over the pelvic area. The model is trained using the annotated data without reviewed in the first place to obtain a corase detector. Later on, we use the annotated data reviewed by superior annotators to train the model again to improve the model’s performance. Table 2 shows the model’s performances with metrics of sensitivity and mFROC. For the record, sensitivity and mFROC are effect evaluating indicator frequently used in image recognition tasks. To be specific, the metric sensitivity is defined as the proportion of all the true lymph nodes detected to all the real lymph nodes annotated. The free-response receiver operating characteristic curve (FROC) describes the variation of the sensitivity as the average number of false-positives (FPs) per CT study changes. Furthermore, the metric mFROC (mean FROC) is defined as the average sensitivity at eight default false-positive rates, which are 0.125, 0.25, 0.5, 1, 2, 4, 8 and 16 FPs per CT study. The higher of the two metrics that the neural network model can achieve, the more accurate the model can obtain. As shown in Table 2, the model without calibration achieved 79% in sensitivity and 35.1% in mFORC. The benchmarks are increased to 83% and 39.1% with calibration. The results indicates the model’s performance got promoted using calibrated data. It implies the expert database of authentic annotated medical images is valuable, for the reviewing process can contribute to the model’s performance. We are now making great efforts to decrease the model’s FP predictions and perform some partition detection research. With the help of TeachMe, it is expected that more incredible method of detecting abdominal lymph nodes will be proposed. What is more, as the technologies of model compression and acceleration such as knowledge distillation^22^ progress, to deploy the detection model on mobile devices can be expected soon.

**Table 2:** The differences in benchmarks with and without the calibration.

Metrics	Without calibration	With calibration
Sensitivity	0.790	0.830
mFROC	0.351	0.391

### Ethical declarations

This article does not contain any studies with human participants or animals performed by any of the authors. The authors declare that all methods were carried out in accordance with relevant guidelines and regulations. All medical images in the study are retrieved from West China Hospital’s picture archiving and communication system (PACS). All experimental protocols were approved by West China Hospital. The study is a retrospective study, which does not involve clinical intervention. All the private and sensitive information of the medical images are eliminated. The Ethics Committee on Biomedical Research, West China Hospital of Sichuan University, has approved that the qualifications of researchers and the research approach meet ethical requirements. Moreover, they have confirmed the ethical approval that exempts this study from the signature of written informed consent.

## Future work

TeachMe is a web-based teaching system for annotating abdominal lymph nodes, easy to access without installation. It provides a three-level annotation-review workflow to collect an expert dataset for automated abdominal lymph nodes research. Also, it is a novel teaching platform for junior doctors to train their interpretation skills via its feedback mechanism. However, there are some objectives that TeachMe should fulfill for improvements: (1) At present, TeachMe doesn’t support DICOM format medical images directly. Medical images demonstrated in TeachMe are with fixed settings like window width, window level. Users can not adjust the brightness and contrast to get the most transparent view for deliberating the tricky regions. Thus, supporting the DICOM format is a demand to improve the experience of annotating. (2) Since we design TeachMe for the detection of abdominal lymph nodes at the outset. The rectangle annotation tool can be completely satisfied for this demand. But when it comes to abdominal lymph nodes segmentation, it is more appropriate to annotate the abdominal lymph nodes with annotation tools like bidirectional cross-shaped annotation tool or free sketch annotation tool. Thus, TeachMe should provide more types of annotation tools for different annotation tasks, which will make the system more flexible. (3) As TeachMe is custom-designed for gastroenterology surgery doctors, the labels set in TeachMe are the classifications of abdominal lymph nodes that the superior annotators formulated and confirmed by all the experts in our medical team. At present, TeachMe is only suitable for abdominal lymph node annotation tasks. If the system enables the superior annotators to formulate customized labels, TeachMe can be ideal for more scenarios.
